# Occupational Characteristics and Management Measures of Sporadic COVID-19 Outbreaks From June 2020 to January 2021 in China: The Importance of Tracking Down “Patient Zero”

**DOI:** 10.3389/fpubh.2021.670669

**Published:** 2021-04-30

**Authors:** Maohui Feng, Qiong Ling, Jun Xiong, Anne Manyande, Weiguo Xu, Boqi Xiang

**Affiliations:** ^1^Department of Gastrointestinal Surgery, Wuhan Peritoneal Cancer Clinical Medical Research Center, Hubei Key Laboratory of Tumor Biological Behaviors, Hubei Cancer Clinical Study Center, Zhongnan Hospital of Wuhan University, Wuhan, China; ^2^Department of Anesthesiology, The Second Affiliated Hospital of Guangzhou University of Chinese Medicine, Guangzhou, China; ^3^Hepatobiliary Surgery Center, Union Hospital of Tongji Medical College, Huazhong University of Science and Technology, Wuhan, China; ^4^School of Human and Social Sciences, University of West London, London, United Kingdom; ^5^Department of Orthopedics, Tongji Hospital of Tongji Medical College, Huazhong University of Science and Technology, Wuhan, China; ^6^School of Public Health, University of Rutgers, New Brunswick, NJ, United States

**Keywords:** COVID-19 disease, severe acute respiratory syndrome coronavirus 2, patient zero, sporadic cases, occupational characteristics, protective measures, person-to-person transmission

## Abstract

There are occupational disparities in the risk of contracting COVID-19. Occupational characteristics and work addresses play key roles in tracking down “patient zero.” The present descriptive analysis for occupational characteristics and management measures of sporadic COVID-19 outbreaks from June to December 2020 in China offers important new information to the international community at this stage of the pandemic. These data suggest that Chinese measures including tracking down “patient zero,” launching mass COVID-19 testing in the SARS-CoV-2-positive areas, designating a new high- or medium-risk area, locking down the corresponding community or neighborhood in response to new COVID-19 cases, and basing individual methods of protection on science are effective in reducing the transmission of the highly contagious SARS-CoV-2 across China.

## Introduction

The transmission of the novel severe acute respiratory syndrome coronavirus 2 (SARS-CoV-2) includes the fomite route through contacts, droplet-borne route transmitted by medium or large droplets, and airborne route through aerosols that can remain suspended over a longer time (1–6). COVID-19 outbreaks not only highlighted the vulnerability of many patients and residents, but also the limited clinical support that led to international headlines. The World Health Organization (WHO) declared a “public health emergency of international concern” and “pandemic of COVID-19” on January 30, 2020 and March 11, 2020, respectively (7–10). By January 26, 2021, more than 100 million COVID-19 cases have been reported in more than 188 countries, resulting in over 2.14 million deaths worldwide.

With much of the world still in the grips of the pandemic COVID-19, importation of the SARS-CoV-2 virus poses a great potential threat to epidemic prevention and control in China. Though China took a number of strict measures to prevent importing the virus *via* inbound travelers and imported goods over the past few months, local sporadic outbreaks of COVID-19 were reported from June 2020 to January 2021. In these new sporadic outbreak regions, detection of “No. 0 source of infection” is crucial work. It is well-known that there are occupational disparities in the risk of contracting COVID-19 (11). “Patient zero” refers to the first human infected by the virus in a new outbreak region, which may be one or more confirmed/asymptomatic cases. Occupational characteristics and work addresses of “patient zero” play key roles in tracking down “No. 0 source of infection” (12). We collected and analyzed data on occupational characteristics and management measures tackling COVID-19 in China.

## Methods

At the beginning of June 1, 2020, we prospectively focus on the COVID-19 epidemic data from the Chinese Center for Disease Control and Prevention every day. Once receiving the new report of a confirmed or asymptomatic case in China, we will track this epidemic, collect its epidemiological characteristics from announcements by the local Health Commission, and present a descriptive analysis for occupational characteristics and management measures of sporadic outbreaks of COVID-19 (from June to December 2020).

Publicly available information was compiled from announcements by local authorities from the China Center for Disease Control and Prevention, the National Health Commission in Tianjin, Beijing, Qingdao, Dalian, Shenyang, Shanghai, and the WHO. We also searched reports from the media and announcements from the local Municipal Health Commissions related to patient zero or cold-chain food transmission infection control from June to January 2021. Also, we collected literature using the PubMed database and Cochrane Library from June 1, 2019 to January 31, 2021. Search terms included “patient zero” and “novel coronavirus” or “COVID-19” or “2019-nCoV”.

## Results

### Occupational Distribution of “Patient Zero” in Local Sporadic Outbreak Regions

**Figure 1:**
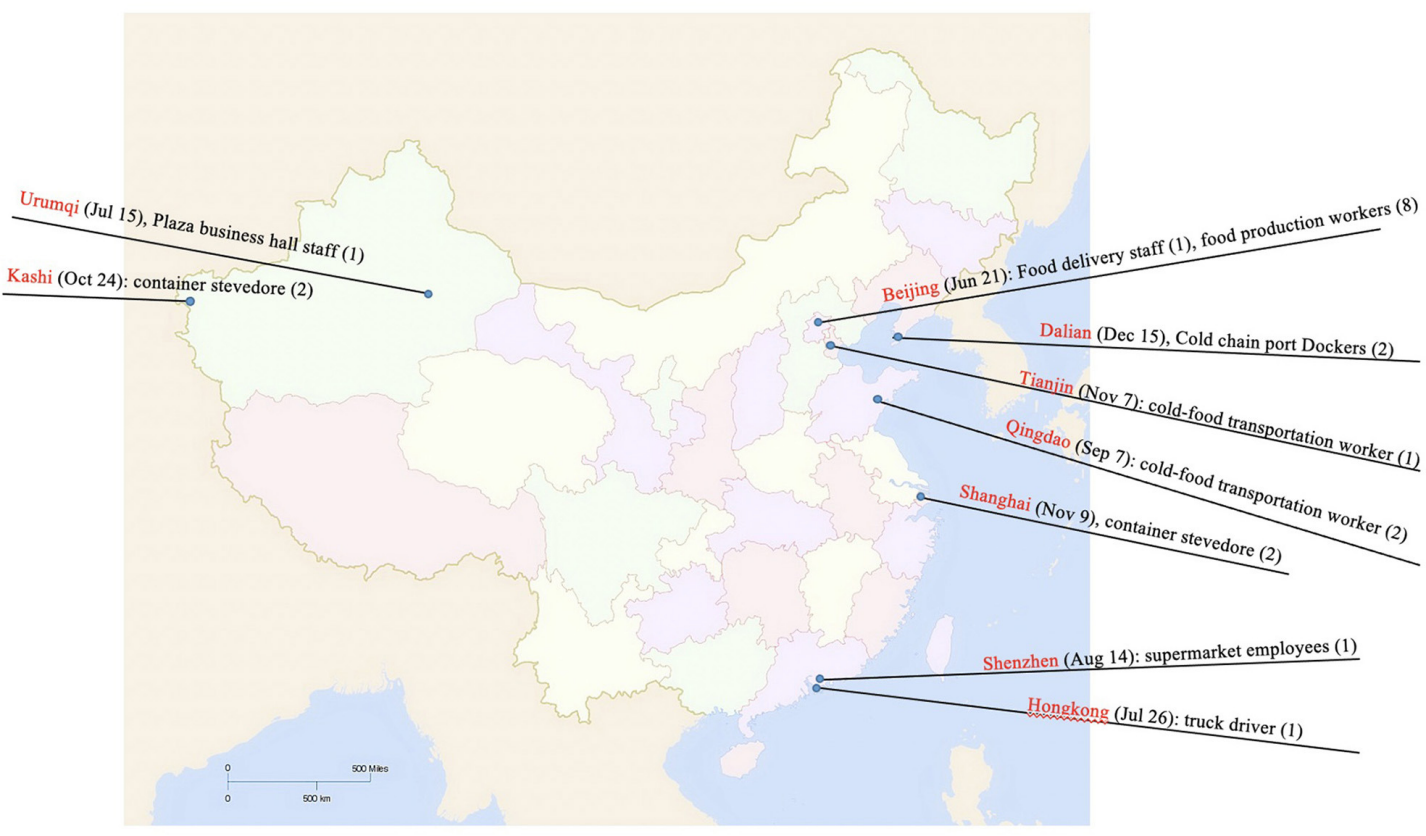
Geographical and occupational distribution of local sporadic outbreak cases with COVID-19 from June to December 2020. Five cities (Dalian, Tianjin, Qingdao, Kashgar, and Shanghai) reported the “patient zero.” For the outbreak in Kashgar and Shanghai, the source of the epidemic was frozen or wet containers outside the border and their “patient zero” was container stevedore who was asymptomatic. Plaza business hall staff (1) in Urumqi (July 15), supermarket employees (1) in Shenzhen (August 14), truck driver (1) in Hong Kong (July 26), Food delivery staff (1) and food production workers (8) in Beijing (June 21) were diagnosed as confirmed cases.

**Figure 2:**
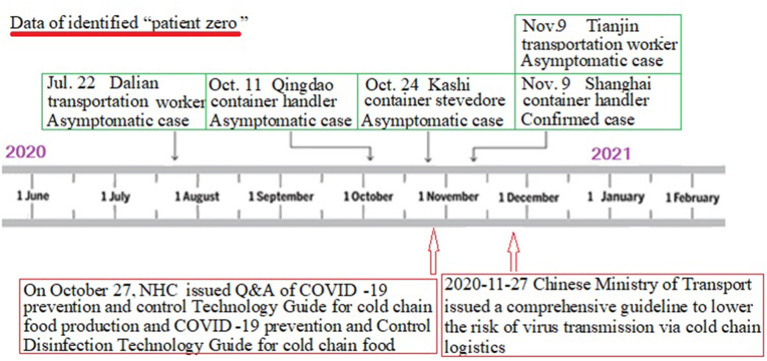
The graph's left-right axis (dates from June to December 2020) is used as a timeline of the key events and dynamic profile of “patient zero” during the local sporadic COVID-19 outbreak. From June to December 2020, five cities in China had reported over nine “patient zero,” including two confirmed cases and seven asymptomatic cases. On October 27, NHC issued Q&A of COVID-19 prevention and control Technology Guide for cold chain food production and COVID-19 prevention and Control Disinfection Technology Guide for cold chain food. The contents include hygiene management measures at the source of cold chain goods, prevention and control requirements in the process of production and processing, prevention and control requirements in the process of sales and operation, cleaning and disinfection operations in the process of cold chain food production and processing and precautions for disinfection of equipment or surfaces of environmental objects. On November 27, the Chinese Ministry of Transport issued a comprehensive guideline to lower the risk of virus transmission *via* cold chain logistics.

Tracing “No. 0 source of infection” is crucial work in new sporadic outbreak regions. From June to December 2020, the occupational distribution of patient zero in local sporadic outbreak regions with COVID-19 is shown in Figure 1. Data of identified “patient zeros” in new sporadic outbreak regions are presented in Table 1. From June to December 2020, five cities in China had reported over nine “patient zeros,” including two confirmed cases and seven asymptomatic cases (Figure 2 and Table 1). These “patient zeros” may be involved in a cold-chain environment-to-human transmission of COVID-19 (Table 1). For the outbreak in Kashgar and Shanghai, the source of the epidemic was frozen or wet containers outside the border and their “patient zero” was container stevedore who was asymptomatic.

**Table 1:** Data of identified “patient zero” in new sporadic outbreak regions.

**City**	**Date**	**Patient zero**	**Occupation**	**Diagnosis**	**Source of infection**
Dalian	July 22	2	Transportation worker	Asymptomatic case	Cold-food
Qingdao	October 11	2	Container handler	Asymptomatic case	Cold seafood
Kashi	October 24	2	Container stevedore	Asymptomatic case	Imported containers
Shanghai	November 9	2	Container handler	Confirmed case	Imported containers
Tianjin	November 9	1	Transportation worker	Asymptomatic case	Cold-food

In addition, plaza business hall staff (1) in Urumqi (July 15), supermarket employees (1) in Shenzhen (August 14), truck driver (1) in Hong Kong (July 26), and Food delivery staff (1) and food production workers (8) in Beijing (June 21) were diagnosed as confirmed cases (Figure 1).

### Detection of SARS-CoV-2 on Imported Frozen Raw Foods and Their Packaging Materials Across China

**Figure 3:**
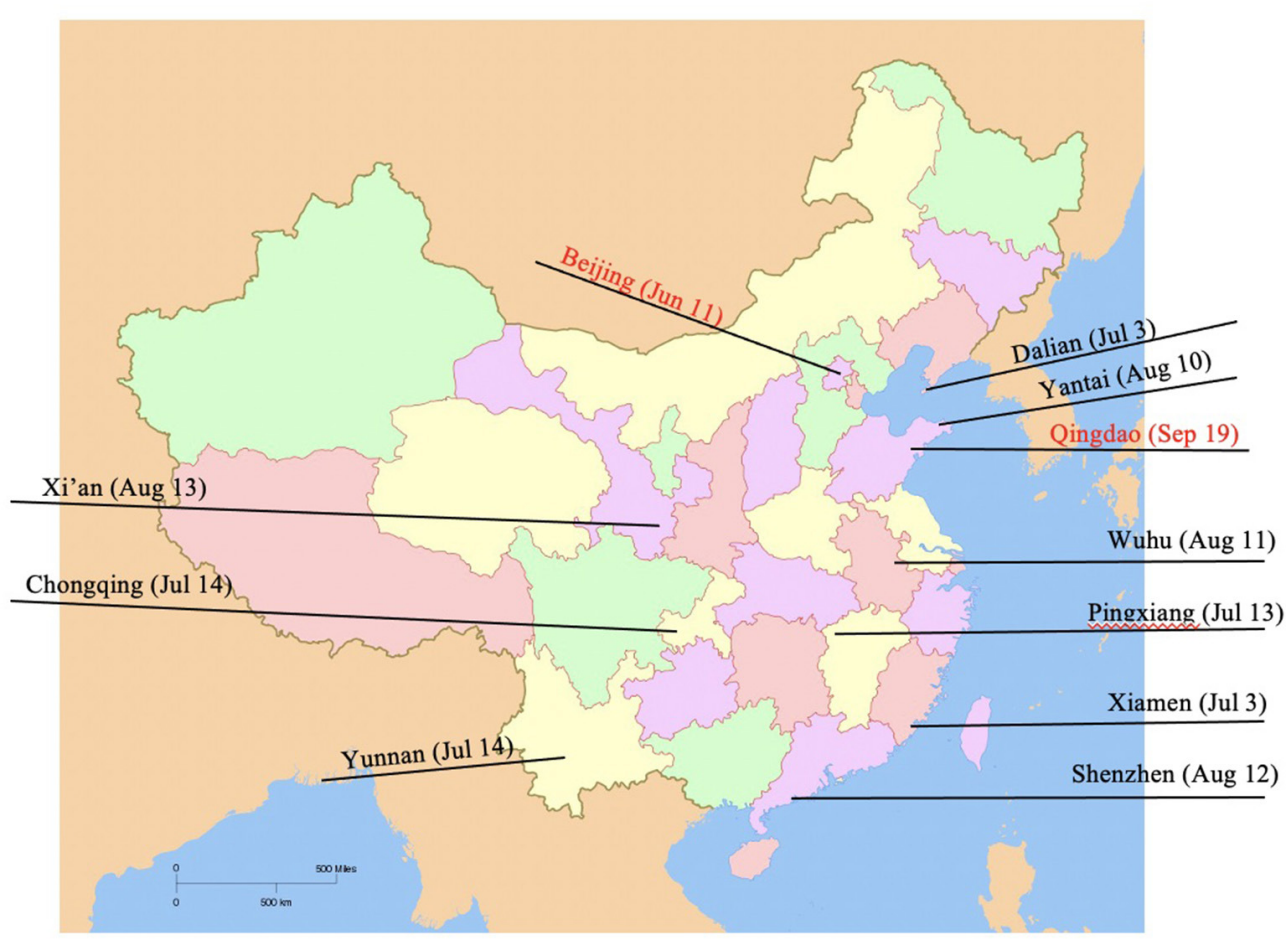
ARS-CoV-2 detection on imported frozen raw foods and their packaging materials across China. During the outbreak in Beijing, five fish swab samples related to imported cold chain food (Salmon) tested positive for SARS-CoV-2 nucleic acid in Xinfadi market. As for the outbreak in Qingdao, the live SARS-CoV-2 virus was successfully isolated from the imported frozen cod package surfaces. The SARS-CoV-2 was also found on frozen food surfaces, packaging materials and food storage environments in many cities, for example Dalian (July 3), Xiamen (July 3) Shenzhen (August 12), etc.

Since the beginning of June 2020, at least 10 incidents of food contamination have been reported across the country, where SARS-CoV-2 was detected on imported frozen raw foods, mostly on their packaging materials (Figure 3). During the outbreak in Beijing, five fish swab samples related to imported cold chain food (Salmon) tested positive for SARS-CoV-2 nucleic acid in the Xinfadi market and the virus genome sequence obtained from one unopened fish swab sample was highly homologous to the virus in human and environmental samples in this epidemic. As for the outbreak in Qingdao, the live SARS-CoV-2 virus was successfully isolated from the imported frozen cod package surfaces.

In addition, the novel coronavirus SARS-CoV-2 was found on frozen food surfaces, packaging materials and food storage environments in many cities, for example Dalian (July 3), Xiamen (July 3) Shenzhen (August 12), etc.

### Epidemiological Characteristics and Occupational Distribution of Sporadic Nosocomial COVID-19 Infections

Epidemiological characteristics and occupational distribution of sporadic nosocomial COVID-19 infections is presented in Table 2. From June 2020 to January 2021, six hospitals in China had reported over 23 confirmed cases. Among them, nursing staff (3), doctors (4), cleaner (1), patients (4) accompanying staff (9), and logistics support staff (2) were diagnosed. The route of infection mainly included workplace accidental exposure to COVID-19, nosocomial infection between healthcare workers and patients, family member exposure with COVID-19, cold-chain environment-to-human transmission and imported from overseas.

**Table 2:** Epidemiological characteristics of sporadic nosocomial COVID-19 infections.

**Date**	**City**	**Number of hospitals**	**Confirmed cases**	**Profession**	**Route of infection**
June 14	Beijing	1	1	Nurse	Family member exposure with COVID-19
October 11	Qingdao	1	6	Patients and accompanying staffs	Nosocomial infection
October 29	Qingdao	1	1	Nurse	Workplace accidental exposure to COVID-19
December 27	Dalian	1	1	Cleaner	Cold-chain environment-to-human transmission
January 3	Shenyang	1	12	Medical staff, patients and accompanying staffs	Nosocomial infection
January 20	Shanghai	2	2	Logistics support staffs	Imported from overseas

## Discussion

A main finding of the occupational characteristics of new sporadic outbreaks with COVID-19 from June 2020 to January 2021 in China was central to tracking down “patient zero.” Here we offered a first description of the occupational distribution of “patient zero,” epidemiological characteristics and the occupational distribution of sporadic nosocomial COVID-19 infections. Chinese management measures including tracking down “patient zero,” launching mass COVID-19 testing in SARS-CoV-2-positive areas, designating a new high or medium-risk area, locking down the corresponding community or neighborhood in response to new COVID-19 cases, and basing individual methods of protection on science were all found to be effective in rapidly reducing transmission of the highly contagious SARS-CoV-2 across China.

Based on the WHO's definition, transmission classifications of the contagious disease include no confirmed cases, sporadic cases, clusters of cases and community transmission. Sporadic cases are defined as cities/areas with one or more cases, imported or locally detected. Clusters of cases refer to cities/areas experiencing cases, clustered in time, geographic location and/or by common exposures. Community transmission are defined as cities/areas experiencing larger outbreaks of local transmission defined through an assessment of factors including, but not limited to: (1) Large numbers of cases not linkable to transmission chains; (2) Large numbers of cases from sentinel lab surveillance; (3) Multiple unrelated clusters in several areas of the country/cities/areas (13). Based on the above analyses, we suggest that SARS-CoV-2 infections in six cities belong to new sporadic outbreaks of COVID-19 from June 2020 to January 2021 in China.

In new sporadic outbreak regions, tracing “patient zero,” also known as the index case, is important work. “Patient zero” refers to the first human infected by the virus in a new outbreak region, which may be one or more confirmed/asymptomatic cases. Tracking down “patient zero” could provide new insights for epidemiologists and local authorities about the nature of first transmissions into a population and to understanding the novel SARS-CoV-2 virus behind the ongoing global health crisis and how to curb further transmission (14). In many hard-hit regions, it was difficult to know how the SARS-CoV-2 virus got there or who contracted it first (15). But in new sporadic outbreak regions, we recommend that identifying “patient zero” may be more useful. Our data showed that five cities in China reported over nine “patient zeros” from June to December 2020.

The potential role of the environment-to-person transmission spread of COVID-19 highlights the importance of detecting “No. 0 source of infection” and tracking down “patient zero.” Jones et al. (16) reported the shedding of SARS-CoV-2 in feces and urine and its potential role in person-to-person transmission and the environment-based spread of COVID-19. Our data showed that imported frozen raw foods and their packaging materials tested positive for SARS-CoV-2 nucleic acid, suggesting that the “No. 0 source of infection” and “patient zero” are involved in cold-chain environment-to-human transmission of COVID-19.

The outbreak of SARS-CoV-2 virus places medical staff at an increased risk of infection as they are in close contact with patients with COVID-19 (17–19). Effective control of SARS-CoV-2 transmission between healthcare workers and patients is important during a period of community prevalence of COVID-19 (18). The data showed that extreme caution of authorities, high-grade protection, and social distancing were crucial strategies of hospital prevention and protection measures (20, 21). Dantes et al. (22) reported that delayed recognition of community transmission of covid-19 resulted in healthcare worker infections. Jones et al. (23) showed that the proportion of both asymptomatic and symptomatic healthcare workers (HCWs) testing positive for SARS-CoV-2 rapidly declined to near-zero between 25th April and 24th May 2020, which corresponded to the decline in patient admissions with COVID-19 during the ongoing UK “lockdown,” suggesting that infection prevention and control measures may help prevent hospitals from becoming independent ‘hubs' of SARS-CoV-2 transmission. Our data indicated that Chinese scientific measures could effectively curb the nosocomial spread of the COVID-19.

In conclusion, the present analysis of occupational characteristics and management measures of sporadic COVID-19 outbreaks from June 2020 to December 2021 in China offers important information to the international community. Chinese scientific measures including tracking down “patient zero” or “No. 0 source of infection,” launching mass COVID-19 testing and individual methods of protection based on science are crucial strategies to curb further spread of COVID-19 and prevent the importation of the SARS-CoV-2 virus *via* inbound travelers and imported goods.

## Data Availability Statement

The original contributions presented in the study are included in the article/supplementary material, further inquiries can be directed to the corresponding author.

## Author Contributions

MF, QL, and JX: data collection. WX, AM, and BX: data interpretation and writing. All authors contributed to the article and approved the submitted version.

## Conflict of Interest

The authors declare that the research was conducted in the absence of any commercial or financial relationships that could be construed as a potential conflict of interest.
